# COPD-derived fibroblasts secrete higher levels of senescence-associated secretory phenotype proteins

**DOI:** 10.1136/thoraxjnl-2020-215114

**Published:** 2020-12-03

**Authors:** Roy R Woldhuis, Irene H Heijink, Maarten van den Berge, Wim Timens, Brian G G Oliver, Maaike de Vries, Corry-Anke Brandsma

**Affiliations:** 1 Pathology and Medical Biology, University Medical Centre Groningen, Groningen, The Netherlands; 2 Groningen Research Institute for Asthma and COPD (GRIAC), University of Groningen, Groningen, The Netherlands; 3 Respiratory Cellular and Molecular Biology Group, Woolcock Institute of Medical Research, Glebe, New South Wales, Australia; 4 School of Life Sciences, University of Technology Sydney, Sydney, New South Wales, Australia; 5 Pulmonary Diseases, University Medical Centre Groningen, Groningen, The Netherlands; 6 Epidemiology, University Medical Centre Groningen, Groningen, The Netherlands

**Keywords:** COPD mechanisms, COPD pathology

## Abstract

COPD-derived fibroblasts have increased cellular senescence. Senescent cell accumulation can induce tissue dysfunction by their senescence-associated secretory phenotype (SASP). We aimed to determine the SASP of senescent fibroblasts and COPD-derived lung fibroblasts, including severe, early-onset (SEO)-COPD. SASP protein secretion was measured after paraquat-induced senescence in lung fibroblasts using Olink Proteomics and compared between (SEO-)COPD-derived and control-derived fibroblasts. We identified 124 SASP proteins of senescent lung fibroblasts, of which 42 were secreted at higher levels by COPD-derived fibroblasts and 35 by SEO-COPD-derived fibroblasts compared with controls. Interestingly, the (SEO-)COPD-associated SASP included proteins involved in chronic inflammation, which may contribute to (SEO-)COPD pathogenesis.

## Introduction

Accelerated lung ageing has been postulated to contribute to the pathogenesis of COPD.^1^ Several mechanisms of accelerated ageing have been identified in COPD,^1 2^ of which cellular senescence is most extensively described to be increased in lung tissue and structural cells from patients with COPD.^3^ Cellular senescence is an irreversible cell cycle arrest that prevents cell death.^4^ Senescent cells secrete (pro-inflammatory) proteins, called the senescence-associated secretory phenotype (SASP), to recruit immune cells for their clearance. However, on accumulation of senescent cells, high levels of SASP proteins can have detrimental effects on the surrounding tissue, by inducing chronic inflammation and tissue dysfunction.^5^ The SASP is cell type specific and its potential (negative) impact on surrounding cells largely depends on the composition and level of secretion of these SASP proteins. Examples of previously described SASP proteins include interleukins, chemokines, growth factors and proteases.^6 7^

Recently, we demonstrated higher levels of cellular senescence in lung fibroblasts and lung tissue from patients with older, mild-moderate COPD and patients with severe, early-onset (SEO)-COPD compared with their matched controls.^8^ Patients with SEO-COPD develop very severe COPD at a relatively early age with relatively low numbers of pack-years. Thus, accelerated lung ageing, including cellular senescence, may contribute to SEO-COPD. The SASP of senescent primary lung fibroblasts and COPD-derived fibroblasts is not defined yet and thus the potential impact of senescent fibroblasts on the surrounding lung tissue is unclear. Therefore, we aimed to first identify SASP proteins of senescent primary human lung fibroblasts and second to determine which of these SASP proteins are secreted at higher levels by COPD-derived fibroblasts, including SEO-COPD, compared with their matched non-COPD control-derived fibroblasts.

## Methods

Cell culture supernatants from lung fibroblasts from 10 patients with SEO-COPD and 11 patients with older, mild-moderate COPD and, respectively, 9 and 10 matched non-COPD controls were used (table 1), which were collected as previously described^8^ (a detailed description of the methods can be found in the online supplemental). Briefly, cellular senescence was induced in fibroblasts from all subject groups by paraquat (PQ) treatment (250 µM for 24 hours), which by occupational exposure is a risk factor for COPD, and can induce senescence specifically via mitochondrial reactive oxygen species production.^9 10^ Senescence induction was confirmed by a 40% increase in SA-β-gal positive cells and a sevenfold increase in p21 expression.^8^ Cell culture supernatants were collected 4 days after senescence induction. The highly sensitive Olink Proteomics (Olink Proteomics, Uppsala, Sweden) panels *Inflammation* and *Cardiovascular III* were used to measure the secretion of 184 proteins, whereof 165 proteins passed quality control. Since cell numbers at the end of culture were significantly different between COPD and control and between PQ and untreated (online supplemental figure S1), levels of secreted proteins were corrected for these cell numbers. Significant differences between PQ treated and untreated cells were tested using Wilcoxon signed-rank test adjusted for multiple testing using Benjamini-Hochberg. Proteins were defined as SASP protein when a significant (FDR<0.05) ≥threefold increase in secretion was observed after PQ treatment. Next, statistical differences in SASP protein secretion between untreated COPD-derived and control-derived fibroblasts were tested using Mann-Whitney U test. FDR p<0.05 was considered statistically significant. Finally, pathway analysis of COPD-associated SASP proteins was performed using the STRING database (V.11.0) to provide more insight into the function of the SASP proteins and their potential role in COPD, while it should be noted that the selected panels may have caused a bias in the analysis.

10.1136/thoraxjnl-2020-215114.supp1 Supplementary data

**Table 1:** Subject characteristics of fibroblasts of combined groups and subgroups

Variable	Control	COPD	P value	Variable	Control (SEO-COPD-matched)	SEO-COPD	P value	Control (older COPD-matched)	Older, mild-moderate COPD	P value
Number	19	21		Number	9	10		10	11	
Age, mean years (range)	61 (42–81)	62 (44–81)	0.844	Age, mean years (range)	52 (42–59)	50 (44–55)	0.349	70 (65–81)	73 (66–81)	0.176
Male/female, n	9/10	12/9	0.548	Male/female, n	1/8	2/8	0.556	8/2	10/1	0.500
Pack-years	34 (28–40)	30 (15–50)	0.627	Pack-years	32 (28–35)	26 (14–30)	0.673	43 (28–51)	49 (19–53)	0.823
Stop-months,	120 (30–240)	78 (36–96)	0.337	Stop-months	84 (18–168)	78 (63–93)	0.677	186 (81–252)	66 (27–96)	0.421
Non-COPD, n	19	–		Non-COPD, n	9	–		10	–	
COPD, n	–	21		COPD, n	–	10		–	11	
GOLD 1	–	–		GOLD 1	–	–		–	–	
GOLD 2	–	7		GOLD 2	–	–		–	7	
GOLD 3	–	4		GOLD 3	–	–		–	4	
GOLD 4	–	10		GOLD 4	–	10		–	–	
FEV_1_ %pred	88.1 (82.5–98.0)	38.8 (17.1–66.7)	**0.000**	FEV_1_ %pred	87.0 (83.5–92.0)	16.5 (14.3–22.7)	**0.000**	90.7 (82.2–104.0)	66.7 (43.4–70.5)	**0.000**
FVC %pred	90.3 (83.0–107.5)	77.9 (44.2–83.5)	**0.005**	FVC %pred	92.8 (84.6–101.0)	42.6 (37.9–68.1)	**0.000**	89.5 (76.7–107.5)	83.5 (79.7–98.8)	0.647
FEV_1_/FVC	73.6 (71.8–77.7)	41.8 (28.4–50.0)	**0.000**	FEV_1_/FVC	75.9 (73.3–79.0)	27.6 (26.0–38.5)	**0.000**	72.1 (70.3–75.1)	50.0 (41.7–59.0)	**0.000**

Data are presented as medians with interquartile ranges unless otherwise stated.

Significant differences between groups were tested using Mann–Whitney U tests or unpaired t-tests. P values are stated.

Gold stage based on FEV_1_ %pred.

%pred, % predicted; SEO, severe, early-onset.

## Results

**Figure 1:**
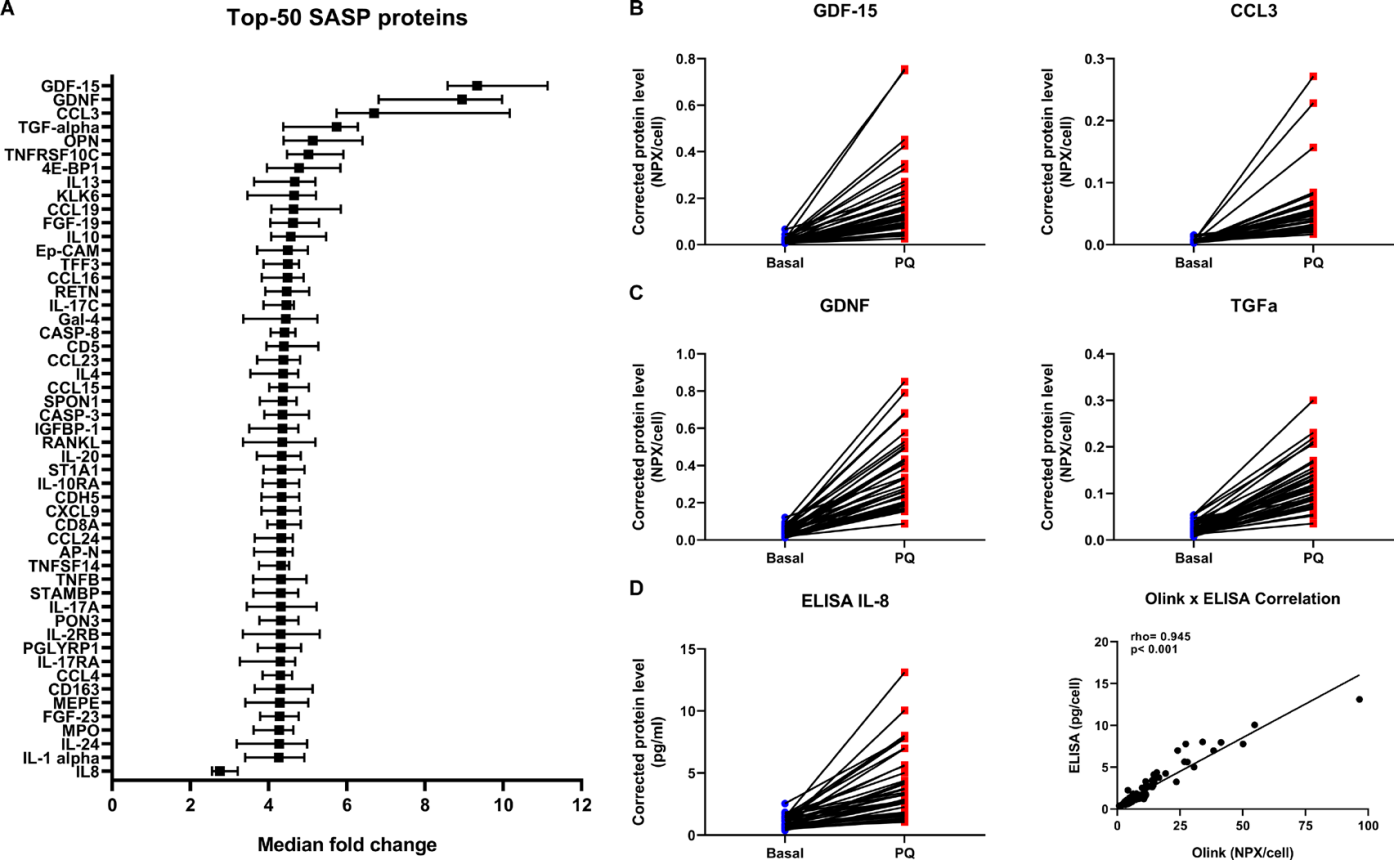
SASP of senescent primary lung fibroblasts. Graph showing top-50 of 124 significant SASP proteins with highest median fold change and IL-8, sorted on fold change (A). Significant differences were tested using Wilcoxon signed-rank tests (n=40). Benjamini-Hochberg adjusted FDR<0.05 was considered statistically significant. Medians with 95% CI are plotted. Examples of two previously described SASP proteins, that is, GDF-15 and CCL3 (B) and two not previously described SASP proteins, that is, GDNF and TGF-α (C) with the highest median fold change are plotted in dot plots (for more details see online supplemental table S1). Blue=basal and red=paraquat (PQ) treatment (both n=40). Protein levels are depicted as Olink NPX values corrected for total cell numbers. IL-8 protein levels were validated using Human DuoSet ELISA (R&D Systems, Abingdon, UK) (D) and correlated with Olink IL-8 levels (D, right panel). Spearman Rho and p value are plotted in the graph. FDR, false discovery rate; IL, interleukin; SASP, senescence-associated secretory phenotype.

First, the secretion of 124 proteins was significantly increased ≥threefold after senescence induction by PQ and these proteins were thus defined as SASP proteins of senescent primary lung fibroblasts (top-50 is shown in figure 1A, see online supplemental table S1 for all SASP proteins). We compared our SASP composition with the recently published SASP Atlas^7^ and other literature and included the overlap in online supplemental table S1. From the 124 found SASP proteins 70 were previously described, including GDF-15 and CCL-3 (figure 1B). In addition, our approach revealed 54 potentially novel SASP proteins, including GDNF and TGF-α (figure 1C). We validated the Olink Proteomics platform by measuring IL-8 using ELISA. A similar increase in IL-8 secretion was detected by ELISA after PQ-induced senescence with a significant positive correlation with IL-8 levels measured by Olink Proteomics (figure 1D).

**Figure 2:**
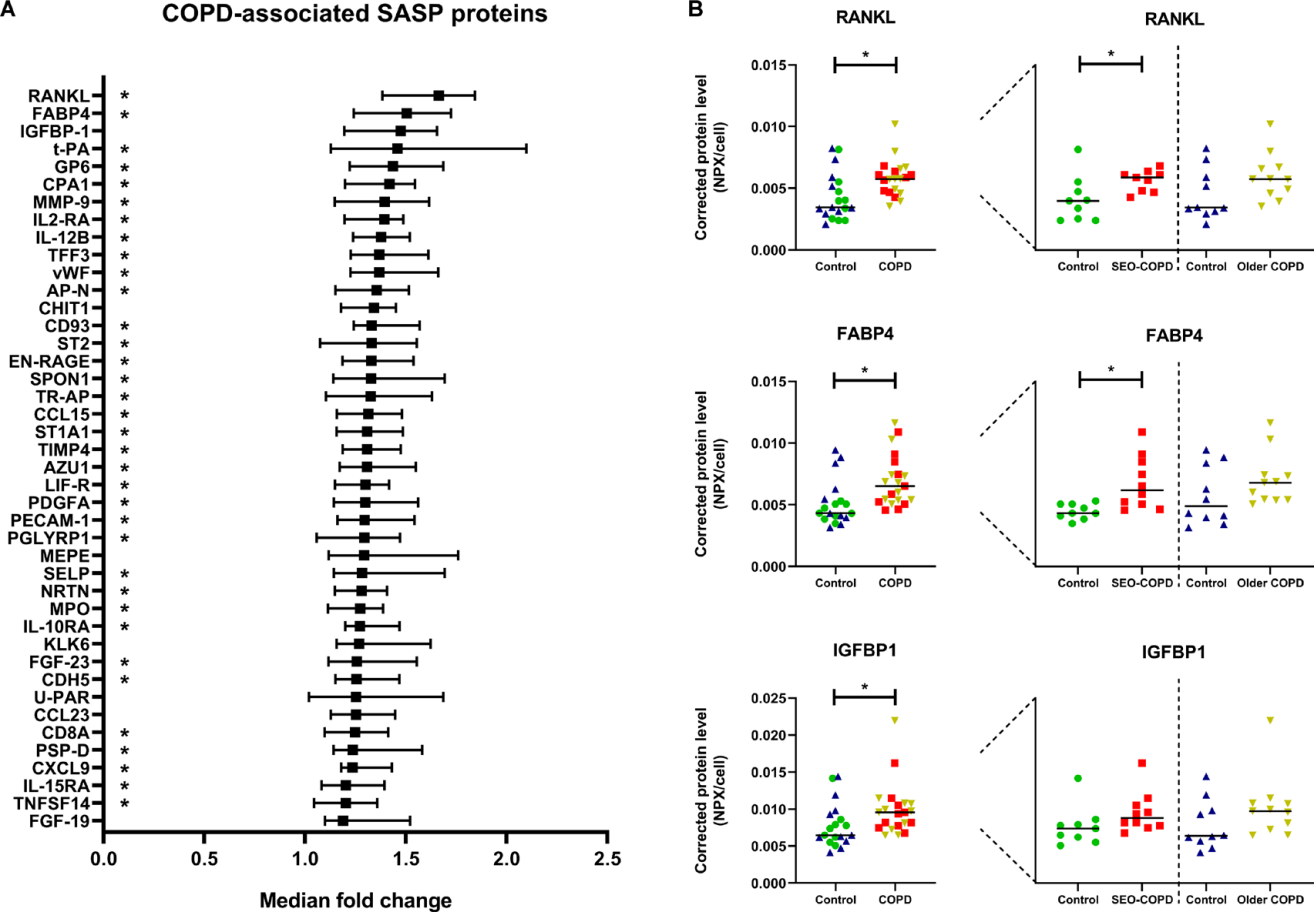
Higher levels of SASP protein secretion by COPD-derived fibroblasts. Graph showing all 42 significant SASP proteins with higher protein secretion in COPD-derived fibroblasts (n=21) compared with non-COPD controls (n=19), sorted on fold change (A) (for more details see online supplemental table S2). Significant differences were tested using Mann-Whitney U tests. Benjamini-Hochberg adjusted FDR<0.05 was considered statistically significant. Medians with 95% CI are plotted. The SEO-COPD-associated SASP proteins are indicated with a star in the graph behind the protein names. No older, mild-moderate COPD-associated SASP proteins were found. The three COPD-associated SASP proteins with the highest fold change in medians are plotted in dot plots (B). Green=SEO-COPD-matched controls (n=9), red=SEO COPD (n=10), blue=older, mild-moderate COPD-matched controls (n=10), yellow=older, mild-moderate COPD (n=11). Protein levels are depicted as Olink NPX values corrected for cell numbers. Lines represent medians. SASP, senescence-associated secretory phenotype; SEO, severe, early-onset. FDR, false discovery rate.

Next, the secreted levels of these 124 defined SASP proteins were evaluated in untreated cell culture supernatants from patients with COPD compared with their matched control-derived fibroblasts. We observed higher levels of 42 SASP proteins in supernatants from COPD-derived fibroblasts (figure 2A, see online supplemental table S2 for a detailed overview). The three proteins with the highest median fold change were RANKL, FABP4 and IGFBP-1 (figure 2B). Several of the COPD-associated SASP proteins were previously found to be higher expressed at the transcription level in COPD-derived lung tissue compared with controls, including vWF, CHIT1, SPON1, TR-AP, TIMP4, PECAM1, CDH5, PSP-D, IL-15RA.^11^ Furthermore, several COPD-associated SASP proteins were associated with ageing in lung tissue at the transcription level, including t-PA, CHIT1, SPON1, IL-10RA and CXCL9.^12^ On subgroup analyses, 35 of the 42 COPD-associated proteins were secreted at higher levels by fibroblasts from patients with SEO-COPD compared with their matched controls (online supplemental table S2), whereas this was not the case for the patients with older, mild-moderate COPD compared with their matched controls.

Finally, STRING pathway analysis revealed that responses to stimuli, immune responses and cytokine-related pathways are associated with the COPD-associated SASP proteins (data not shown). COPD-associated SASP proteins include cytokines (IL12B, TNFSF14 and RANKL) and chemokines (CCL15, CCL23 and CXCL9) that are known to be involved in inflammatory processes. These findings suggest that the SASP proteins that are secreted at higher levels by COPD-derived fibroblasts might be involved in the chronic inflammatory response in COPD.

## Conclusion

By using a proteomic-based approach, we provide insight into the SASP of primary human lung fibroblasts. Interestingly, 42 of the 124 identified SASP proteins were secreted at higher levels by fibroblasts from patients with COPD compared with matched controls. The COPD-associated SASP proteins include proteins that have been implicated in chronic inflammation, and thus may contribute to disease pathology in COPD. Remarkably, 35 of these 42 COPD-associated SASP proteins are secreted at higher levels by patients with SEO-COPD compared with their matched controls, whereas none were significantly different between patients with older, mild-moderate COPD compared with their matched controls. This lack of significance is likely due to higher biological variation in these older subgroups as the fold changes are comparable (online supplemental table S2) and the interquartile ranges are higher in these groups (online supplemental figure S2). These results suggest a role for these SASP proteins in COPD. The fact that both cellular senescence and SASP protein secretion were higher in COPD-derived lung fibroblasts compared with their matched controls suggests that senescence accumulation is involved in the pathogenesis of COPD. It should be noted that until now it is unknown whether the higher senescence observed in COPD is driven by acute exposures or chronic exposures, which may result in a different SASP profile. In addition, different senescence-inducing stimuli may result in a different SASP profile as well. The identified (COPD-associated) SASP proteins of primary lung fibroblasts can be used for further studies to understand the role of senescent cell accumulation and its potential detrimental impact in SEO-COPD pathogenesis.
