# Supplementary material for: COPD-derived fibroblasts secrete higher levels of senescence-associated secretory phenotype proteins
Source: Thorax. 2020 Dec 3;76(5):508–11. doi: 10.1136/thoraxjnl-2020-215114 (PMC8070616; doi:10.1136/thoraxjnl-2020-215114)
Supplement: Supplementary data [file thoraxjnl-2020-215114supp001.pdf]

COPD-derived fibroblasts secrete higher levels of senescence-associated secretory phenotype proteins

Online Supplement

Complete methods

Subjects

Primary lung fibroblasts from subjects undergoing lung transplantation or tumour resection surgery were used. Resected lung tissue was isolated distal from the tumour and was macroscopically and histologically normal. Primary parenchymal lung fibroblasts were isolated and cultured as described before [1]. Briefly, parenchymal lung tissue was cut into small cubes and cultured in 12-wells plates in Ham's F12 medium supplemented with 10% foetal calf serum (FCS), 2mM L-Glutamine, 100µg/ml Streptomycin and 100U/ml penicillin at 37°C and 5% CO_2. Medium was refreshed every week and after four weeks fibroblasts were trypsinized and placed into 25 cm^2 flasks. When cultures reached confluency, fibroblasts were frozen and stored in liquid nitrogen. The following inclusion criteria were used:

1) SEO-COPD patients; FEV1/FVC <70% and FEV1 <30%pred measured at an age <53 (according to [2]) and with age <56 at time of lung transplant surgery
2) non-COPD control subjects (SEO-COPD-matched); FEV1/FVC >70%, age <60 at time of surgery
3) Older, mild-moderate, COPD patients; FEV1/FVC <70% and FEV1 30-80%pred, age >65 at time of surgery
4) non-COPD control subjects (Older COPD-matched); FEV1/FVC >70%, age >65 at time of surgery

None of the COPD patients was alpha-1 antitrypsin deficient. To get sufficient SEO-COPD-matched non-COPD control subjects, subjects at an age <60 at the time of surgery were included, taken into account the age-matching with the SEO-COPD group.

The study protocol was consistent with the Research Code of the University Medical Centre Groningen and national ethical and professional guidelines ("Code of conduct; Dutch federation of biomedical scientific societies", htttp://www.federa.org). Lung fibroblasts and lung tissues used in this study are derived from left-over lung material after lung surgery and transplant procedures. This material was not subject to the act on medical research involving human subjects in the Netherlands and therefore an ethics waiver was provided by the Medical Ethical Committee of the University Medical Centre Groningen (METc UMCG). All samples and clinical information were de-identified before experiments were performed.

Primary parenchymal lung fibroblast culture

The primary parenchymal lung fibroblasts were defrosted and cultured in batches of four, including fibroblasts from each subgroup in equal numbers, as described before [1]. At passage 5, 25000 fibroblasts were seeded in Ham's F12 medium + 5% FCS in 12-well plates and after two days treated with or without 250 µM Paraquat dichloride hydrate (PQ) (Sigma-Aldrich, Zwijndrecht, the Netherlands) for 24 hours to induce cellular senescence [3]. After 24 hours, PQ was removed and cells were kept in culture for four days in Ham's F12 medium + 5% FCS. These time-points were carefully chosen based on pilot study results.

Olink Proteomics

The highly sensitive Olink Proteomics (Olink Proteomics, Uppsala, Sweden) panels *Inflammation* and *Cardiovascular III*, were used to measure the secretion of 184 proteins, whereof 165 proteins passed QC. The Olink Proteomics analysis uses an antibody-based method called Proximity Extension Assay technology. Briefly, oligonucleotide-labelled antibody pairs bind the target protein and when oligonucleotides are in close proximity, these hybridize and get extended by a DNA polymerase. This created DNA barcode is amplified and quantified by qPCR. A full explanation about this analysis can be found on their website: https://www.olink.com/data-you-can-trust/technology/. Levels of secreted proteins were corrected for total cell numbers four days after senescence induction.

Secreted protein analyses

Cell-free supernatants were harvested four days after PQ removal and stored in -80°C prior to analyses. Secreted IL-8 levels were measured using Human DuoSet ELISA (R&D Systems, Abingdon, United Kingdom). As the numbers of cells were different at the end of culture between COPD and control-derived fibroblasts, and between untreated and PQ-treated, we corrected the secreted protein levels for cell numbers counted at the end of culture.

Statistical analyses

SPSS software was used for the statistical analyses. Significant differences between PQ treated and untreated cells were tested using Wilcoxon signed-rank test adjusted for multiple testing using Benjamini-Hochberg. Proteins were defined as SASP protein when a significant (FDR <0.05) ≥3-fold increase in secretion was observed upon PQ treatment. Next, statistical differences in SASP protein secretion between untreated COPD- and control-derived fibroblasts were tested using Mann-Whitney U. FDR P<0.05 was considered statistically significant.

Table S1: Overview of all 124 defined SASP proteins

PROTEIN	FOLD CHANGE	P-VALUE	FDR	DESCRIBED OR NOVEL?
GDF-15	9.331	5.255E-08	7.226E-08	SASP Atlas
GDNF	8.946	5.255E-08	7.226E-08	Potentially novel
CCL3	6.695	5.683E-08	7.442E-08	SASP Atlas
TGF-ALPHA	5.737	5.255E-08	7.226E-08	Potentially novel
OPN	5.129	5.255E-08	7.226E-08	Potentially novel
TNFRSF10C	5.017	5.253E-08	7.226E-08	Previously described
4E-BP1	4.776	5.255E-08	7.226E-08	SASP Atlas
IL13	4.668	5.255E-08	7.226E-08	Previously described
KLK6	4.651	5.253E-08	7.226E-08	Potentially novel
CCL19	4.636	5.253E-08	7.226E-08	SASP protein family
FGF-19	4.621	5.253E-08	7.226E-08	SASP protein family
IL10	4.564	5.255E-08	7.226E-08	Previously described
EP-CAM	4.490	9.008E-07	1.047E-06	Potentially novel
TFF3	4.486	5.255E-08	7.226E-08	Potentially novel
CCL16	4.484	5.255E-08	7.226E-08	Previously described
RETN	4.459	5.255E-08	7.226E-08	Potentially novel
IL-17C	4.455	5.255E-08	7.226E-08	Previously described
GAL-4	4.435	5.255E-08	7.226E-08	Potentially novel
CASP-8	4.409	5.255E-08	7.226E-08	Potentially novel
CD5	4.387	5.255E-08	7.226E-08	Potentially novel
CCL23	4.379	5.253E-08	7.226E-08	SASP protein family
IL4	4.373	5.255E-08	7.226E-08	Previously described
CCL15	4.370	5.253E-08	7.226E-08	SASP protein family
SPON1	4.359	5.255E-08	7.226E-08	Potentially novel
CASP-3	4.351	5.253E-08	7.226E-08	Previously described
IGFBP-1	4.350	5.255E-08	7.226E-08	SASP protein family
RANKL	4.346	5.255E-08	7.226E-08	Potentially novel
IL-20	4.335	5.255E-08	7.226E-08	SASP protein family
ST1A1	4.332	5.255E-08	7.226E-08	Potentially novel
IL-10RA	4.331	5.255E-08	7.226E-08	SASP protein family
CDH5	4.330	5.255E-08	7.226E-08	Potentially novel
CXCL9	4.328	5.255E-08	7.226E-08	SASP protein family
CD8A	4.322	5.253E-08	7.226E-08	Potentially novel
CCL24	4.321	5.255E-08	7.226E-08	SASP protein family
AP-N	4.320	5.255E-08	7.226E-08	SASP Atlas
TNFSF14	4.316	5.255E-08	7.226E-08	SASP protein family
TNFB	4.316	5.255E-08	7.226E-08	SASP protein family
STAMBP	4.311	5.253E-08	7.226E-08	Potentially novel
IL-17A	4.309	5.253E-08	7.226E-08	Previously described
PON3	4.309	5.255E-08	7.226E-08	Potentially novel
IL-2RB	4.308	5.255E-08	7.226E-08	SASP protein family
PGLYRP1	4.305	5.255E-08	7.226E-08	Potentially novel
IL-17RA	4.302	5.255E-08	7.226E-08	SASP protein family

CCL4	4.301	5.255E-08	7.226E-08	SASP protein family
CD163	4.301	5.255E-08	7.226E-08	Potentially novel
MEPE	4.287	5.255E-08	7.226E-08	Potentially novel
FGF-23	4.278	5.251E-08	7.226E-08	SASP protein family
MPO	4.271	5.255E-08	7.226E-08	Previously described
IL-24	4.269	5.255E-08	7.226E-08	SASP protein family
IL-1 ALPHA	4.262	3.782E-07	4.588E-07	Previously described
PSP-D	4.249	5.255E-08	7.226E-08	Potentially novel
CCL28	4.247	5.255E-08	7.226E-08	SASP protein family
SELP	4.239	5.255E-08	7.226E-08	Potentially novel
LIF-R	4.225	5.253E-08	7.226E-08	Potentially novel
TNFRSF14	4.224	5.255E-08	7.226E-08	SASP protein family
VWF	4.217	5.255E-08	7.226E-08	Potentially novel
SIRT2	4.214	5.253E-08	7.226E-08	Potentially novel
AZU1	4.212	5.253E-08	7.226E-08	Potentially novel
FGF-21	4.211	5.255E-08	7.226E-08	SASP protein family
CD6	4.190	5.255E-08	7.226E-08	Potentially novel
MMP-9	4.183	5.255E-08	7.226E-08	SASP Atlas
CCL25	4.182	5.255E-08	7.226E-08	Previously described
SCGB3A2	4.179	5.253E-08	7.226E-08	Potentially novel
TR	4.175	5.253E-08	7.226E-08	SASP Atlas
CPA1	4.172	5.253E-08	7.226E-08	Potentially novel
CD244	4.168	5.255E-08	7.226E-08	Potentially novel
PECAM-1	4.166	5.255E-08	7.226E-08	Potentially novel
TNF	4.166	5.251E-08	7.226E-08	Previously described
NOTCH 3	4.159	5.253E-08	7.226E-08	Potentially novel
IL-22 RA1	4.153	5.255E-08	7.226E-08	SASP protein family
OSM	4.151	5.251E-08	7.226E-08	Potentially novel
TR-AP	4.141	5.255E-08	7.226E-08	Potentially novel
IL-20RA	4.129	5.255E-08	7.226E-08	SASP protein family
IL-1RT2	4.125	5.255E-08	7.226E-08	SASP protein family
EN-RAGE	4.121	4.070E-07	4.831E-07	Potentially novel
NRTN	4.114	5.255E-08	7.226E-08	Potentially novel
IL2	4.105	5.253E-08	7.226E-08	Previously described
ADA	4.097	5.253E-08	7.226E-08	Potentially novel
IFN-GAMMA	4.095	5.255E-08	7.226E-08	Previously described
U-PAR	4.093	5.255E-08	7.226E-08	SASP Atlas
ICAM-2	4.090	5.255E-08	7.226E-08	Potentially novel
AXIN1	4.089	5.255E-08	7.226E-08	Potentially novel
TIMP4	4.081	5.253E-08	7.226E-08	SASP protein family
CHIT1	4.078	5.255E-08	7.226E-08	Potentially novel
CPB1	4.068	5.255E-08	7.226E-08	Potentially novel
GP6	4.050	5.255E-08	7.226E-08	Potentially novel
ARTN	4.048	5.255E-08	7.226E-08	Potentially novel
VEGFA	4.047	5.255E-08	7.226E-08	Previously described
IL18	4.025	9.669E-07	1.101E-06	SASP Atlas

DNER	4.018	5.255E-08	7.226E-08	Potentially novel
TSLP	3.994	5.255E-08	7.226E-08	Potentially novel
IL33	3.989	5.255E-08	7.226E-08	SASP protein family
IL5	3.985	5.255E-08	7.226E-08	SASP protein family
PDGFA	3.950	5.255E-08	7.226E-08	Previously described
SHPS-1	3.948	5.255E-08	7.226E-08	Potentially novel
CD93	3.944	5.253E-08	7.226E-08	Potentially novel
ST2	3.938	5.255E-08	7.226E-08	SASP protein family
IL2-RA	3.912	5.253E-08	7.226E-08	SASP protein family
LTBR	3.896	5.255E-08	7.226E-08	Potentially novel
PCSK9	3.847	5.255E-08	7.226E-08	Potentially novel
SELE	3.833	5.251E-08	7.226E-08	Potentially novel
IL-18BP	3.785	5.255E-08	7.226E-08	SASP protein family
IL-15RA	3.781	5.255E-08	7.226E-08	SASP protein family
EPHB4	3.756	5.253E-08	7.226E-08	Potentially novel
TNFRSF9	3.736	5.255E-08	7.226E-08	SASP protein family
TLT-2	3.680	5.255E-08	7.226E-08	Potentially novel
FABP4	3.667	5.255E-08	7.226E-08	Previously described
NT-PROBNP	3.666	5.255E-08	7.226E-08	Potentially novel
GAL-3	3.548	5.253E-08	7.226E-08	SASP Atlas
CX3CL1	3.547	5.683E-08	7.442E-08	Previously described
BETA-NGF	3.487	5.255E-08	7.226E-08	Previously described
IL-10RB	3.474	5.255E-08	7.226E-08	SASP protein family
SCF	3.449	5.255E-08	7.226E-08	Previously described
CCL20	3.442	1.196E-06	1.351E-06	Previously described
IL-18R1	3.440	5.255E-08	7.226E-08	SASP protein family
T-PA	3.424	5.683E-08	7.442E-08	SASP Atlas
CXCL11	3.302	5.255E-08	7.226E-08	Previously described
TNF-R2	3.263	5.253E-08	7.226E-08	Previously described
IL-12B	3.259	5.253E-08	7.226E-08	SASP protein family
PD-L1	3.166	5.255E-08	7.226E-08	Potentially novel
CTSZ	3.100	5.255E-08	7.226E-08	SASP Atlas
FGF-5	3.042	5.255E-08	7.226E-08	SASP protein family
CXCL16	3.029	5.255E-08	7.226E-08	SASP protein family
CD40	3.011	4.070E-07	4.831E-07	Previously described

- *Fold change: Median of fold changes between PQ treated and untreated primary lung fibroblasts.*
- *P-value: tested using Wilcoxon signed-rank tests.*
- *FDR: P-value adjusted for multiple testing using Benjamini-Hochberg correction.*
- *Last column describes overlap with SASP Atlas [4], PubMed search for previous described, and protein families of described SASP proteins [5].*

Table S2: Overview of all 42 COPD associated SASP proteins

	COPD VS CONTROLS			SEO-COPD VS MATCHED CONTROLS			OLDER, MM COPD VS MATCHED CONTROLS		
**PROTEIN**	**FC**	**P-value**	**FDR**	**FC**	**P-value**	**FDR**	**FC**	**P-value**	**FDR**
**RANKL**	1.6630	0.0054	**0.0379**	1.4804	0.0193	**0.0379**	1.6704	0.0783	0.1820
**FABP4**	1.5049	0.0018	**0.0379**	1.4286	0.0071	**0.0375**	1.3885	0.1809	0.2111
**IGFBP-1**	1.4742	0.0113	**0.0450**	1.1928	0.0500	0.0568	1.5197	0.0910	0.1820
**T-PA**	1.4575	0.0132	**0.0450**	1.6420	0.0114	**0.0375**	1.1056	0.3981	0.4180
**GP6**	1.4362	0.0051	**0.0379**	1.4196	0.0179	**0.0379**	1.4577	0.0910	0.1820
**CPA1**	1.4189	0.0011	**0.0379**	1.3138	0.0033	**0.0375**	1.4426	0.0573	0.1820
**MMP-9**	1.3942	0.0122	**0.0450**	1.5136	0.0043	**0.0375**	1.1451	0.4813	0.4931
**IL2-RA**	1.3935	0.0030	**0.0379**	1.3822	0.0143	**0.0375**	1.3723	0.0573	0.1820
**IL-12B**	1.3780	0.0070	**0.0379**	1.5486	0.0243	**0.0379**	1.2342	0.1590	0.1964
**TFF3**	1.3685	0.0076	**0.0379**	1.2113	0.0338	**0.0443**	1.4524	0.0671	0.1820
**VWF**	1.3678	0.0076	**0.0379**	1.3410	0.0222	**0.0379**	1.2822	0.1590	0.1964
**AP-N**	1.3555	0.0047	**0.0379**	1.3294	0.0222	**0.0379**	1.4039	0.0573	0.1820
**CHIT1**	1.3418	0.0047	**0.0379**	1.2939	0.0500	0.0568	1.4140	0.0411	0.1820
**CD93**	1.3304	0.0008	**0.0379**	1.2934	0.0143	**0.0375**	1.3839	0.0242	0.1820
**ST2**	1.3296	0.0030	**0.0379**	1.4317	0.0114	**0.0375**	1.1581	0.1213	0.1960
**EN-RAGE**	1.3288	0.0169	**0.0500**	1.3847	0.0305	**0.0427**	1.2412	0.2050	0.2265
**SPON1**	1.3282	0.0076	**0.0379**	1.3119	0.0222	**0.0379**	1.3587	0.0910	0.1820
**TR-AP**	1.3258	0.0055	**0.0379**	1.3555	0.0338	**0.0443**	1.3781	0.0783	0.1820
**CCL15**	1.3137	0.0060	**0.0379**	1.2205	0.0275	**0.0412**	1.3429	0.0910	0.1820
**ST1A1**	1.3074	0.0024	**0.0379**	1.4220	0.0118	**0.0375**	1.3526	0.0671	0.1820
**TIMP4**	1.3072	0.0070	**0.0379**	1.2771	0.0143	**0.0375**	1.3158	0.1590	0.1964
**AZU1**	1.3067	0.0039	**0.0379**	1.3340	0.0143	**0.0375**	1.2909	0.1213	0.1960
**LIF-R**	1.3004	0.0145	**0.0450**	1.3056	0.0152	**0.0375**	1.3207	0.1213	0.1960
**PDGFA**	1.2988	0.0033	**0.0379**	1.3515	0.0412	**0.0495**	1.4048	0.0346	0.1820
**PECAM-1**	1.2950	0.0036	**0.0379**	1.2810	0.0179	**0.0379**	1.5383	0.0671	0.1820
**PGLYRP1**	1.2943	0.0097	**0.0445**	1.4064	0.0222	**0.0379**	1.2557	0.1590	0.1964
**MEPE**	1.2928	0.0132	**0.0450**	1.3013	0.0864	0.0864	1.2737	0.0783	0.1820
**SELP**	1.2824	0.0036	**0.0379**	1.3386	0.0114	**0.0375**	1.2940	0.0783	0.1820
**NRTN**	1.2803	0.0064	**0.0379**	1.1821	0.0305	**0.0427**	1.4130	0.0573	0.1820
**MPO**	1.2742	0.0097	**0.0445**	1.2814	0.0143	**0.0375**	1.3080	0.1392	0.1964
**IL-10RA**	1.2725	0.0059	**0.0379**	1.2351	0.0118	**0.0375**	1.3135	0.1053	0.1960
**KLK6**	1.2679	0.0122	**0.0450**	1.4624	0.0604	0.0634	1.1514	0.1590	0.1964
**FGF-23**	1.2570	0.0157	**0.0474**	1.2329	0.0118	**0.0375**	1.3974	0.2313	0.2491
**CDH5**	1.2547	0.0036	**0.0379**	1.2409	0.0090	**0.0375**	1.4702	0.0910	0.1820
**U-PAR**	1.2530	0.0142	**0.0450**	1.4439	0.0864	0.0864	1.2140	0.0486	0.1820
**CCL23**	1.2522	0.0076	**0.0379**	1.2071	0.0576	0.0621	1.2669	0.0486	0.1820
**CD8A**	1.2480	0.0124	**0.0450**	1.2829	0.0380	**0.0469**	1.2830	0.1590	0.1964
**PSP-D**	1.2365	0.0122	**0.0450**	1.6285	0.0025	**0.0375**	1.0308	0.5262	0.5262
**CXCL9**	1.2358	0.0145	**0.0450**	1.2279	0.0380	**0.0469**	1.3673	0.1590	0.1964
**IL-15RA**	1.2023	0.0124	**0.0450**	1.2376	0.0243	**0.0379**	1.1905	0.2050	0.2265

**TNFSF14**	1.2016	0.0114	**0.0450**	1.1898	0.0243	**0.0379**	1.2558	0.1809	0.2111
**FGF-19**	1.1882	0.0145	**0.0450**	1.1634	0.0576	0.0621	1.3390	0.1213	0.1960

- *FC (Fold change): Fold change in medians of COPD compared to control-derived fibroblasts.*
- *P-value: tested using Mann-Whitney U tests.*
- *FDR: P-value adjusted for multiple testing using Benjamini-Hochberg correction. Boldfaced when significant.*

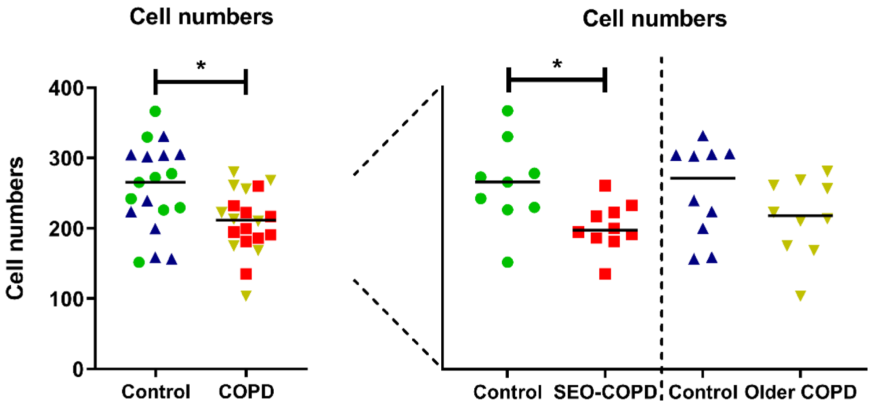

Figure S1: Cell number differences between fibroblasts from COPD patients and controls at baseline. *Dot plots show total cell number at the end of culture of all 4 patient groups. Green = SEO-COPD-matched control, red = SEO-COPD, blue = older COPD-matched control, yellow = older, mild-moderate, COPD. Lines represent medians. Significant differences tested with Mann-Whitney U tests. * P-value < 0.05.*

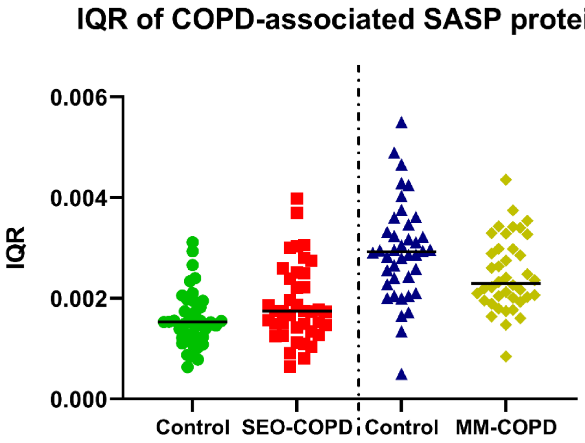

Figure S2: Interquartile ranges of COPD-associated proteins per subgroup*. Interquartile ranges (IQR) of the 42 COPD-associated SASP proteins per subgroup. Green = SEO-COPD-matched controls, red = SEO-COPD, blue = older, mild-moderate COPD-matched controls, yellow = older, mild-moderate COPD.*

References

1 Noordhoek JA, Postma DS, Chong LL*, et al*. Different modulation of decorin production by lung fibroblasts from patients with mild and severe emphysema. *COPD.* 2005;**2** (suppl 1):17-25.

2 Silverman EK, Chapman HA, Drazen JM*, et al*. Genetic epidemiology of severe, early-onset chronic obstructive pulmonary disease. Risk to relatives for airflow obstruction and chronic bronchitis. *Am J Respir Crit Care Med.* 1998;**157** (suppl 6 Pt 1):1770-1778.

3 Chinta SJ, Woods G, Demaria M*, et al*. Cellular Senescence Is Induced by the Environmental Neurotoxin Paraquat and Contributes to Neuropathology Linked to Parkinson's Disease. *Cell Rep.* 2018;**22** (suppl 4):930-940.

4 Basisty N, Kale A, Jeon OH*, et al*. A proteomic atlas of senescence-associated secretomes for aging biomarker development. *PLoS Biol.* 2020;**18** (suppl 1):e3000599.

5 Coppe JP, Desprez PY, Krtolica A*, et al*. The senescence-associated secretory phenotype: the dark side of tumor suppression. *Annu Rev Pathol.* 2010;**5**:99-118.
